# Introduction to a generalized method for adaptive randomization in trials

**DOI:** 10.1186/1745-6215-14-19

**Published:** 2013-01-17

**Authors:** Zoë SJ Hoare, Christopher J Whitaker, Rhiannon Whitaker

**Affiliations:** 1 NWORTH Bangor Clinical Trials Unit, Institute of Medical & Social Care Research, Y Wern, Holyhead Road, George Site, Bangor University, Bangor, LL57 2PZ, GwyneddUK

## Abstract

**Background:**

Ideally clinical trials should use some form of randomization for allocating participants to the treatment groups under trial. As an integral part of the process of assessing the effectiveness of these treatment groups, randomization performed well can reduce, if not eliminate, some forms of bias that can be evident in non-randomized trials. Given the vast set of possible randomization methods to choose from we demonstrate a method that incorporates many of the advantages of these other methods.

**Methods:**

A step-by-step introduction of how to use the adaptive randomization algorithm for conducting a clinical trial is given.

**Results:**

The implications, effects and capabilities of using the adaptive randomization algorithm are fully demonstrated and explained using simulated data and examples from actual trials.

**Conclusions:**

This paper provides an introduction to a dynamic type of treatment allocation, which fulfills the CONSORT requirements of participants being randomly allocated whilst maintaining a level of control of the balances overall, within the stratification variables and within the strata simultaneously. Maintaining control of the imbalances within the groups is vital particularly if interim analyses are planned.

**Trial registration:**

Current controlled trials ISRCTN17551624, ISRCTN37558856, ISRCTN97185214.

## Background

The CONSORT statement says that: ‘Ideally, participants should be assigned to comparison groups in the trial on the basis of a chance (random) process characterized by unpredictability’ [1]. There are many treatment allocation methods available in the literature to achieve this. These vary from simple randomization through to deterministic methods, with other methods showing varying degrees of control of the randomization process [2].

Deterministic methods obviously do not fulfill the requirements denoted in the CONSORT statement and are not now recommended. Simple randomization may be the obvious method to ensure complete randomness of allocations. However, a drawback is that balance within treatment groups cannot be controlled and so imbalance of treatment allocation can occur. Imbalances can cause greater problems in smaller trials (<100 participants) than for larger trials; however, an imbalance of allocated treatments within certain subgroups of the sample can still cause problems within larger trials. Allocation methods to overcome this problem have been developed. These methods exert some control of the randomization process while maintaining unpredictability. Many trials recruit participants sequentially and therefore the issue of maintaining unpredictability whilst retaining a level of control can become more difficult.

Stratification variables are variables chosen because it is believed they have an effect on the efficacy of the treatment under scrutiny. For example, gender could be treated as a stratification variable, to ensure that there was a balance of genders between the treatment groups. It would not be good if say all male participants were allocated to one treatment group. If there is more than one stratification variable then their combination needs to be considered in assessing the balance in treatment allocation. With two binary stratification variables, for instance, gender and age (<40, ≥40), then there are four strata, one of these being male and <40. If only a few members of the population are sampled in one of these four strata, then it is likely that an imbalance will occur within this stratum.

The method we present here maintains unpredictability during sequential participant recruitment while simultaneously allowing control over the balance across treatment groups, within stratification levels and within strata [3]. Exerting control of the imbalance at the multiple levels of the randomization process simultaneously has not been proposed previously. We think that achieving this with a simple function is the major advantage of this method.

The method has several advantages. It can accommodate two or more treatment groups. It utilizes a dynamic method of calculating allocation probabilities. That is, the probability of allocation to each treatment group is recalculated for every participant based on the participants already allocated. It can accommodate as many discrete leveled stratification variables as desired. It controls balance at the overall level, within stratification variables and within strata, as well as being controlled sequentially. This means that the group balance is kept in check throughout the recruitment and randomization process making interim analyses easily possible. It measures the imbalance at the overall level, at the stratification variable level and at the stratum level, and uses a simple weighted sum of squared differences to combine the imbalances at each level.

## Methods

### The idea and design of the method

**Figure 1:**
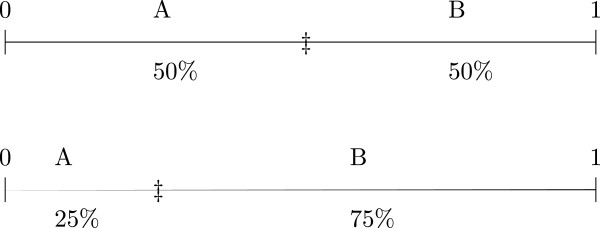
**Probability boundaries for a two group randomization.** First group is a 1:1 allocation to either group at a point where there are equal numbers in each group. The second is an example of a 1:3 allocation in favour of group B.

Figure 1 demonstrates the ideas behind how the participants are allocated. Each time a new participant needs to be randomized to the trial then the boundaries (represented by *†*) between the groups are recalculated. This recalculation takes into account: (a) the number of people already assigned to the treatment groups; (b) the number of people within the relevant stratification level and (c) the number of people within the relevant stratum. The position of the boundaries is adjusted accordingly. Once the boundaries have been calculated, a number between 0 and 1 is randomly generated and the participant is assigned to the group in which the generated number falls.

Weights (or parameters) are attached to each level of the allocation under consideration (that is overall, stratification level and stratum level) to control how much an imbalance affects the movement of the boundary. With larger weights the movements of the proabbility boundaries are more pronounced. If balance at a particular level is considered important then larger weights may be assigned.

This paper explains the method by introducing the base model, a worked example of the method, a selection of simulations to demonstrate the effect of the weights on the algorithm and some examples from recent clinical trials.

### The base method

The method for calculating the boundary between two treatment groups is now given. These four steps form the basis of all randomization set-ups for two or more groups: 

1. Calculate the allocation odds, $\left(\frac{A}{B}\right)$. This is related to the allocation ratio defined as *A*:*B*, that is the number assigned to *A* in relation to *B*. For example, if the intended allocation ratio is 1:1 then the allocation odds are $\left(\frac{1}{1}=1\right)$ and if the intended allocation ratio is 2:1 in favor of *A* then the allocation odds are $\left(\frac{2}{1}=2\right)$.

2. Calculate the difference as:

$$d_{\text{level}}=\sqrt{\text{Allocation odds}}\times \text{Total in}\;B-\frac{\text{Total in}\;A}{\sqrt{\text{Allocation odds}}}$$

at each relevant level (overall, within stratification variable and within strata).

3. Calculate a weighted sum of squared signed differences from Step 2. Take the differences from Step 2, square them, multiply the squared differences by their signs, (0 if the difference was zero, −1 if the difference was negative and 1 if the difference was positive), then multiply these squared signed differences by their relevant weights. Finally add them all up to give a total measure of imbalance.

4. Take the logit of this weighted sum to give the probability of treatment A being assigned. The logit function is:

$$\frac{\text{Allocation odds}\times \exp(a)}{1+\text{Allocation odds}\times \exp(a)}$$

where exp(*a*) is the exponential of the number *a*.

#### Base method example (two-group randomization)

Take a trial with two treatment groups *A* and *B*, with an allocation ratio of 2:1. We shall take two stratification variables: gender (male and female) and center (X, Y and Z). Let the first participant who requires randomization be female and from center Y. Take the weights to be 0.5 for stratum, 0.2 for center and gender and 0.1 for total. 

1. The allocation ratio *A*:*B* is 2:1 therefore the allocation odds are $\left(\frac{2}{1}=2\right)$.

2. Calculate the difference at each relevant level: 

(a) At the overall level we currently have no participants assigned giving:

$$d_{\text{total}}=\sqrt{2}\times 0-\frac{0}{\sqrt{2}}=0$$

(b) At the center level (center Y) we currently have no participants assigned giving:

$$d_{\text{center}}=\sqrt{2}\times 0-\frac{0}{\sqrt{2}}=0$$

(c) At the gender level (female) we currently have no participants assigned giving:

$$d_{\text{gender}}=\sqrt{2}\times 0-\frac{0}{\sqrt{2}}=0$$

(d) At the stratum (female, center Y) level we currently have no participants assigned giving:

$$d_{\text{stratum}}=\sqrt{2}\times 0-\frac{0}{\sqrt{2}}=0$$

3. Take the weighted sum of the signed differences. This is:

$$0.1\times 0^{2}\times 0+0.2\times 0^{2}\times 0+0.2\times 0^{2}\times 0+0.5\times 0^{2}\times 0=0$$

4. Take the logit function of this weighted difference. The probability of assigning the participant to treatment *A* is:

$$\frac{2\times \exp(0)}{1+2\times \exp(0)}=\frac{2}{3}$$

This is the expected result because for the first allocation the participant should have twice as much chance of being assigned to treatment *A* than to treatment *B*. A random number between 0 and 1 is generated. If the random number is less than $\left(\frac{2}{3}\right)$ the participant is assigned to treatment *A*, else the participant is assigned to treatment *B*.

Consider now that 12 participants have been randomized into the trial as shown in Table 1. If the 13th participant to enter the trial was female and from center Z then the probability boundary would be calculated as:

1. As before the allocation ratio is 2:1 therefore the allocation odds are 2.

2. Calculate the difference at each relevant level: 

(a) At the overall level we currently have eight participants in treatment *A* and four in treatment *B* already assigned giving:

$$d_{\text{total}}=\sqrt{2}\times 4-\frac{8}{\sqrt{2}}=0$$

(Using a calculator may not give the exact answer of zero.)

(b) At the center level (center Z) we currently have three participants in treatment *A* and one in treatment *B* giving:

$$d_{\text{center}}=\sqrt{2}\times 1-\frac{3}{\sqrt{2}}=-\frac{1}{\sqrt{2}}$$

(c) At the gender level (female) we currently have four participants assigned to treatment *A* and two assigned to treatment *B* giving:

$$d_{\text{gender}}=\sqrt{2}\times 2-\frac{4}{\sqrt{2}}=0$$

(d) At the stratum (female, center Z) level we currently have two participants assigned to treatment *A* and no participants assigned to treatment *B* giving:

$$d_{\text{stratum}}=\sqrt{2}\times 0-\frac{2}{\sqrt{2}}=-\frac{2}{\sqrt{2}}$$

3. Take the weighted sum of the signed differences. This is:

$$\begin{array}{l} 0.1\times 0^{2}\times 0+0.2\times {\left(\frac{1}{\sqrt{2}}\right)}^{2}\times -1+0.2\times 0^{2}\times 0+0.5\\ \;\;\;\times {\left(\frac{2}{\sqrt{2}}\right)}^{2}\times -1=-1.1 \end{array}$$

4. Take the logit function of this weighted difference. The probability of assigning the participant to treatment *A*:

$$\frac{2\times \exp(-1.1)}{1+2\times \exp(-1.1)}=0.40$$

**Table 1:** 12 allocated participants in the base method example

**Gender**	**Center**	**Treatment*A***	**Treatment*B***
M	X	1	0
F	X	1	1
M	Y	2	1
F	Y	1	1
M	Z	1	1
F	Z	2	0
Totals		8	4

This indicates that the probability of assigning participant 13 to treatment *A* is 0.4 and the probability of assigning them to treatment *B* is 0.6. That means that the boundary has shifted slightly in favor of assigning to treatment *B*. Overall we can see that the assignments are balanced in the required 2:1 ratio (8:4) and also balanced within gender (4:2); however, within the current relevant stratum (center Z and female) there is a current assignment of 2:0 and for center Z there is a current assignment of 3:1, in both cases too many are allocated to *A*. These imbalances result in the higher likelihood of assigning the 13th participant to treatment *B*.

### Algorithm weighting

The weights chosen for use in Step 3 of the base equations determine how important an imbalance at the relevant level is. The bigger the weight used the more an imbalance will be corrected. Generally, the method would be weighted so that stratum has the largest weight, then the stratification variables with middle weight, then the overall weighting having the smallest weight. This is because the number of participants in the stratum (male, center X) would be less than the number of participants in either stratification variable (center or gender) and both of these will be less than the number of participants in the whole of the trial. Imbalances in the smaller numbers have the greater impact and therefore need greater control necessitating the use of larger weights.

Take the first example of the two-group randomization with allocation ratio 2:1. The weights used were 0.5 for the stratum, 0.2 for both the stratification variables and 0.1 for overall. At Step 3 this gave us a weighted imbalance sum of −1.1 giving the probability of assigning to treatment *A* as 0.40.

If the weights are strengthened on this imbalance to 5 for the stratum, 2 for both the stratification variables and 1 for overall, we get a weighted imbalance of:

$$\begin{array}{l} 1\times 0^{2}\times 0+2\times {\left(\frac{1}{\sqrt{2}}\right)}^{2}\times -1+2\times 0^{2}\times 0+5\\ \;\;\;\times {\left(\frac{2}{\sqrt{2}}\right)}^{2}\times -1=-11 \end{array}$$

Taking the logit function of this weighted difference, the probability of assigning the participant to treatment *A*:

$$\frac{2\times \exp(-11)}{1+2\times \exp(-11)}=0.0000167$$

The weights here are extreme, the likelihood of now assigning to treatment *A* is very small. Weights of this strength would not generally be used for a sequential randomization but are used here to illustrate the point that when the weights are increased the imbalance is given more importance and assignment to the underrepresented class becomes more likely.

If the weights are weakened on this imbalance to 0.05 for the stratum, 0.02 for both the stratification variables and 0.01 for overall, we get a weighted imbalance of:

$$\begin{array}{l} 0.01\times 0^{2}\times 0+0.02\times {\left(\frac{1}{\sqrt{2}}\right)}^{2}\times -1+0.022\times 0^{2}\times 0\\ \;\;\;+0.05\times {\left(\frac{2}{\sqrt{2}}\right)}^{2}\times -1=-0.11 \end{array}$$

Taking the logit function of this weighted difference leads to the probability of assigning the participant to treatment *A*:

$$\frac{2\times \exp(-0.11)}{1+2\times \exp(-0.11)}=0.64$$

This probability is barely different from the probability of 0.66 that would be obtained with simple randomization. The imbalances seen within this example are not extreme enough to warrant a huge shift in the calculated probability of assigning to treatment *A*. The weights are used to control the imbalance. If all the weights in the model are set to 0 then the algorithm will perform like simple randomization. If all the weights are set to 10 then this mimics a deterministic randomization with a block size of 2.

## Results and discussion

### Simulations

The reason for developing this method is its ability to control imbalance while preventing predictability.

Simulations were run to show the predicted outcome of a randomization set-up with various sets of weights. Each of 50 randomly generated participants were sequentially allocated to a trial. This was repeated 1,000 times for each set of weights. Take the example from the previous section: a two-group randomization on a 1:1 allocation ratio with two stratification variables, center (X, Y, Z) and gender (male, female). The sets of weights for each scenario are given in Table 2.

**Table 2:** Weights for each simulation scenario

**Scenario**	**Control**	**Overall**	**Center**	**Gender**	**Stratum**
1	strong	1	2	2	5
2	medium	0.1	0.2	0.2	0.5
3	weak	0.01	0.02	0.02	0.05
4	simple randomization	0	0	0	0

From Tables 3, 4 and 5 we can see the control that the algorithm instills. Scenario 1 shows that with the larger weights in control, there is a high likelihood that the final split of allocations will be close to the required split (although not guaranteed). Scenario 3 shows that there is still some control of the allocations in that the worst split obtained is of the order 20–30, which is better than had there been no control as seen with the simple randomization.

**Table 3:** Overall allocated splits of the 50 participants to the two-group trial over the 1,000 simulations

**Group*A***	**Group*B***	**Strong**	**Medium**	**Weak**	**Simple**
					**randomization**
<20	>30				60
20	30			1	55
21	29			3	49
22	28			39	72
23	27		14	113	97
24	26	128	231	205	112
25	25	737	511	249	106
26	24	135	230	234	105
27	23		14	116	96
28	22			38	77
29	21			2	64
30	20				41
> 30	< 20				66

**Table 4:** Imbalances seen within gender across the 1,000 simulations

**Difference**^**a**^	**Strong**	**Medium**	**Weak**	**Simple randomization**
<−7				117
−7			5	56
−6			20	76
−5			46	101
−4		3	110	105
−3		44	165	157
−2	74	214	217	126
−1	506	466	278	172
0	829	543	304	167
1	514	467	264	154
2	77	222	231	169
3		39	184	151
4		2	105	114
5			49	94
6			19	68
7			3	32
>7				141

^a^A difference of −7 indicates that there were seven more participants allocated to treatment group *B* than treatment group *A*.

**Table 5:** Imbalances seen within center across the 1,000 simulations

**Difference**^**a**^	**Strong**	**Medium**	**Weak**	**Simple randomization**
<−8				44
−8			2	38
−7			2	39
−6			21	68
−5			61	104
−4		2	145	93
−3		53	243	158
−2	83	300	349	172
−1	720	706	423	199
0	1372	892	480	201
1	750	684	442	265
2	75	309	347	212
3		47	253	217
4		7	147	148
5			65	125
6			11	87
7			9	78
8				40
>8				54

^a^A difference of −7 indicates that there were seven more participants allocated to treatment group *B* than treatment group *A*.

Tables 6 and 7 indicate the levels of predictability of the allocation sequences. In the strong control scenario we can see from Table 7 that 68% of the probability boundaries calculated are either above 0.90 or below 0.10. This indicates that this implementation of the algorithm is too predictable. It is likely once there is an imbalance that the boundaries will swing widely to rectify the imbalance as soon as possible. The weak control scenario demonstrates that the resulting allocations are limited, that is while the worst split you can expect to achieve is 20–30 (instead of the desired 25–25) this comes at no cost in the predictability of the method and only a tiny percentage of the boundaries calculated were actually less then 0.10 or greater than 0.90. So even if the assessors knew all the weights and the current allocations and how to calculate the boundaries they would still not have a definite knowledge of the treatment group that the next participant would be allocated to.

**Table 6:** Maximum sequences of same group allocations

**Maximum repeated**	**Strong**	**Medium**	**Weak**	**Simple**
**allocation**^**a**^				**randomization**
2	18			
3	509	119	29	22
4	426	445	247	155
5	46	312	307	281
6	1	103	231	215
7		18	118	146
8		3	38	94
9			21	43
10			4	27
11			2	8
12			3	2
13				2
14				2
16				3

^a^In Scenario 1, 1/1000 runs had a maximum run of 6. That is, six allocations to the same treatment group in a row.

**Table 7:** **Probability distributions of the calculated boundaries**^*a*^

**Interval**	**Strong**^**b,c**^	**Medium**^**b**^	**Weak**^**b**^
[0, 0.05]	17184	2275	6
	34%	5%	<1%
(0.05, 0.15]	2506	3394	233
	5%	7%	<1%
(0.15, 0.25]	0	4157	1055
	0%	8%	2%
(0.25, 0.35]	2795	4367	2963
	6%	9%	6%
(0.35, 0.45]	0	5921	8372
	0%	12%	17%
(0.45, 0.55]	5121	10181	25155
	10%	20%	50%
(0.55, 0.65]	0	5897	8112
	0%	12%	16%
(0.65, 0.75]	2887	4188	2852
	6%	8%	6%
(0.75, 0.85]	0	4125	1015
	0%	8%	2%
(0.85, 0.95]	2363	3365	231
	5%	7%	<1%
(0.95, 1]	17144	2130	6
	34%	4%	<1%

^a^Simple randomization has not been included here as the boundary is always at 0.5.

^b^The frequency (%) of the calculated boundaries that occured in each of the intervals is shown.

^c^For the strong control scenario all boundaries in the (0.45, 0.55] interval are 0.5.

An additional advantage of running simulations like these is that various time points may be looked at in relation to the randomization. Table 8 shows the expected result once 12 participants have been randomized to the trial. This information gives us a quick checkpoint to see where the randomization result is in relation to the expected result in real time.

**Table 8:** Allocation splits after 12 of the 50 participants expected have been randomized for each scenario

**Group*A***	**Group*B***	**Strong**	**Medium**	**Weak**	**Simple**
					**randomization**
1	11				1
2	10			3	15
3	9			16	54
4	8		9	98	106
5	7	124	225	248	201
6	6	746	512	264	226
7	5	130	239	245	201
8	4		15	105	115
9	3			19	55
10	2			2	22
11	1				3
12	0				1

Simulations with results like those given in Tables 3 to 8 allow the randomization to be thoroughly tested before the start of recruitment and will give an indication of the outcome that can be expected in advance. This allows real-time tracking of how the method is performing in relation to its expected ranges.

#### Two-group randomization in practice: FolATED equal allocation

FolATED was a HTA-funded multi-centered randomized placebo-controlled trial investigating whether folic acid (folate) would improve patients’ responses to antidepressant treatment and reduce the symptoms of moderate to severe depression [4].

Randomization for the trial was performed remotely by NWORTH using this technique. Participants were to be stratified by: (1) center (Swansea, Wrexham or Bangor); (2) gender (male/female); (3) patient type (new/continuing (that is having taken the same daily antidepressant for at least two months with a stable dose in the therapeutic range (BNF) for at least one month)); (4) the type of antidepressant prescribed (SSRI/other) and (5) whether or not they have ever received counseling for depression.

For system purposes, stratification variables 3 and 5 were combined from two binary stratification variables into one stratification variable with four levels called patient type (new/counseling, new/no counseling, continuing/counseling and continuing/no counseling). It was intended to randomize 549 participants for the trial.

From the simulations the weights for the trial were chosen to be: overall =0.02, center =0.04, gender =0.04, patient type =0.02, type of antidepressant =0.02 and stratum =0.05. The most likely split given this level of weighting was 274:275 (or 275:274) with a probability of 0.64 of this occurring. The most extreme overall split that should have been seen was 272:277 (or 277:272) with a probability of 0.05. An exact balance within the various stratification variables should be seen with a probability of between 0.37 to 0.45. It is likely that the balance within the stratification variables would not have been far beyond the exact balance given the weights chosen.

The number of participants recruited to the trial was 440. Overall 223 participants were allocated to the folate treatment and 217 to the placebo treatment. The folate:placebo allocations by center were: Bangor (110:113), Swansea (56:51), Wrexham (57:53); by gender were female (144:136), male (79:81); by patient type were new (56:52), continuing (167:165); by antidepressant were SSRI (157:145), other (66:72) and by counseling were received (101:97), no (122:120).

#### Two-group randomization in practice: EPIC unequal allocation

EPIC was a pragmatic randomized control trial, designed to evaluate an individually tailored, age-appropriate information pack to support decision-making and self-care relating to insulin management and electronic glucose monitoring for children/young people aged 6–18 years with type 1 diabetes, compared with available resources in routine clinical practice [5]. The target sample size was 252 children/young people with type 1 diabetes. A 2:1 randomization strategy was employed and the intention was to randomize 168 children/young people into the intervention arm and 84 children/young people into the no intervention arm, stratified by age (6–10 years, 11–15 years and 16–18 years), gender and length of time since diagnosis (<2 years and ≥2 years).

Simulations were run and it was decided that the parameter set should be: overall =0.05, gender =0.05, age =0.05, diagnosis =0.05, center =0.1 and stratum =0.2. These simulations indicated that the probability of an overall split of 168:84 would be 0.41, with the worst possible splits being 165:87 (probability 0.01) and 170:82 (probability 0.13).

The EPIC trial actually randomized 339 participants in total. This was balanced overall as 225:114. If the simulations had been run for 339 participants with the selected weights the probability of getting this overall split would have been 0.33. Within gender the balance achieved for male and female was (105:55) and (120:59), respectively. Within the age groups the balance achieved for 6–10 years, 11–15 years and 16–18 years was (79:40), (104:53) and (42:21), respectively. Within diagnosis the balance achieved for less than 2 years and 2 years or more was (48:26) and (177:88), respectively.

#### Three-group randomization in practice: SWAD

SWAD (staying well after depression) was a multi-center, randomized controlled trial [6]. Participants were randomized between mindfulness-based cognitive therapy (MBCT) in addition to treatment as usual (TAU), cognitive psycho-education (CPE) in addition to TAU, and TAU alone in a ratio of 2:2:1. The participants were stratified by: (a) research center (Oxford or Bangor), (b) cohort (six cohorts of recruitment), (c) history of suicidality (none, ideation or suicidal attempt) and (d) whether or not they were taking antidepressants in the 7 days before their first assessment (AD use). Randomization was undertaken by email to the remote randomization center at NWORTH. For validation the randomization email also includes additional information including the participant’s date of birth, gender and date of assessment. The target sample size was 375 randomized participants.

Simulations were run to select the ideal set of weights to use for the randomization system. It was decided to use: overall =0.05, center =0.1, cohort =0.1, antidepressant use =0.05, suicidality =0.05 and stratum =0.25. The exact split of 150:150:75 has a probability of 0.17. However, acceptable results of 149:150:76, 149:151:75, 150:149:76 combined with the exact split have a probability of 0.55. No overall split seen in the simulation was too far away from the intended 2:2:1 ratio.

The SWAD trial actually randomized 276 participants. Overall this was split 110:110:56. From the simulations this was expected with a probability of 0.18. Within center the achieved balance was Bangor (49:49:25) and Oxford (61:61:31). Within antidepressant use the achieved balance was no AD use (62:60:32) and yes AD use (48:50:24). Within suicidality the achieved balance was no suicidality (20:21:12), suicidal ideation (56:55:28) and suicide attempt (34:34:16).

## Conclusions

The randomization method introduced here may appear complex but allows the control of imbalance at various levels of the trial without compromising the predictability. This ultimately allows balanced groups with no increased risk of subversion introduced at any level. The system has been implemented successfully as a web-based randomization system. This system allows approved users to log in and securely enter their data resulting in an instant randomization result. This result can either be blinded or open dependent on the requirements of the trial. A generic system can be implemented allowing any of the situations described earlier. Validation of the data input should also ensure that the data entered is correct – no participant can be randomized twice and there will be no out-of-range dates of birth. This method has been shown to work in a number of fully randomized trials.

## Abbreviations

AD: Antidepressant; CPE: Cognitive psycho-education; MBCT: Mindfulness-based cognitive therapy; SWAD: Staying well after depression.

## Competing interests

The authors declare they have no competing interests.

## Authors’ contributions

All authors contributed to the design of the study and commented on drafts of the manuscript. Zoë Hoare developed, modified and operationalized the theory and drafted this manuscript. Chris Whitaker guided the statistical method from theory to practice and Rhiannon Whitaker directed and resourced the work, and embedded the technique in trials. All authors read and approved the final manuscript.
